# The Central Executive Mediates the Relationship Between Children’s Approximate Number System Acuity and Arithmetic Strategy Utilization in Computational Estimation

**DOI:** 10.3389/fpsyg.2018.00943

**Published:** 2018-06-22

**Authors:** Hongxia Li, Mingliang Zhang, Xiangyan Wang, Xiao Ding, Jiwei Si

**Affiliations:** ^1^School of Psychology, Shandong Normal University, Jinan, China; ^2^Shandong Academy of Governance, Jinan, China

**Keywords:** approximate number system (ANS) acuity, quantile regression, strategy utilization, working memory (WM), Iran-Nejad’s wholetheme spiral of biofunctional understanding

## Abstract

Studies investigating the relationship between working memory (WM) and approximate number system (ANS) acuity in the area of arithmetic strategy utilization are scarce. The choice/no choice method paradigm was used in the present study to determine whether and how ANS acuity and WM components affected strategy utilization. The results showed that the central executive (CE) mediated the relationship between ANS acuity and strategy utilization. Furthermore, quantile regression analyses revealed that the association between CE and strategy choice was robust from the first to highest quantile. Notably, the relationship between ANS acuity and strategy choice was significant at the median and higher quantiles (i.e., 0.5, 0.75, and 0.85 quantiles), but not significant at lower quantiles (i.e., 0.15 and 0.25 quantiles). These results suggest that domain-general skills play a crucial role in the relationship between children’s ANS acuity and mathematical ability. The impact of ANS acuity and CE on strategy choice was dependent on the distribution of the strategy utilization level. These results provide a further understanding of the utilization of cognitive strategies.

## Introduction

Mathematics plays an important role in our daily and professional lives (Hibbard et al., 2007). A long tradition of research in the field of mathematical cognition shows that children use several strategies to accomplish arithmetic problems just like adults (e.g., Siegler and Lemaire, 1997; Siegler, 2007). A strategy is a procedure or a set of procedures for achieving a higher-level goal or task (Lemaire and Reder, 1999). Siegler and Lemaire (1997) proposed a four-dimensional theoretical framework to explain how individuals utilize strategy, including strategy repertoire, strategy distribution, strategy selection, and strategy execution. In this study, we focused mainly on strategy choice and execution aspects.

### The Link Between Working Memory, Approximate Number System Acuity, and Mathematical Performance

Previous studies have revealed that children’s poor performance on mathematics is related to inefficient strategy utilization (e.g., Zhang et al., 2014; Si et al., 2016), making it relevant and essential to explore the factors affecting strategy utilization. Many factors contribute to children’s strategy utilization, including domain-general skills, such as working memory (WM) (Steel and Funnell, 2001), as well as domain-specific skills, such as the acuity of their underlying approximate number system (ANS) (e.g., Sasanguie et al., 2013; Gilmore et al., 2013; and so on).

Working memory has been consistently shown to be an important predictor of children’s math performance. In the last few decades, numerous studies have shown the relationship between WM and strategy utilization. Baddeley (1986) defined WM as a system for temporary retention and manipulation of information while performing a series of cognitive tasks. Baddeley’s model subsequently gained wide acceptance. This model proposes that WM consists of a phonological loop (PL; “limited capacity, speech-based store of verbal information”), a visuo-spatial sketchpad (VS; “response to short-term storage of visual and spatial information, such as memory for an object and their location”), the central executive (CE), and the episodic buffer (Baddeley and Hitch, 1974; Baddeley, 2000). The CE component, which is the core of WM, has a supervisory role in which it regulates and controls the cognitive processes run by the PL and VS. Prior studies have focused on testing the relationship between CE components (i.e., inhibition, switching, updating, and dual-task coordination) and strategy use. For instance, one study revealed that CE load interferes with use of a mental computation strategy (Imbo and Vandierdonck, 2007). Another study revealed that inhibition and shifting mediates age-related differences in strategy selection (Hodzik and Lemaire, 2011). Other studies have demonstrated that dual-task coordination influences estimation efficiency for strategy execution (Ai et al., 2016; Huang et al., 2016). These studies suggest that both CE and VS affect arithmetic performance. However, whether the PL also affects strategy utilization and the differences in the effects of different WM components on strategy use remain to be determined.

Many studies have tested the relationship between the ANS and mathematical ability (e.g., Fuhs and McNeil, 2013; Peng et al., 2017). ANS refers to a system with which an individual can represent a set of magnitudes roughly without calculation, with a fuzziness that increases linearly with the size of the numbers involved (Weber’s law) (Pica et al., 2004). This system is shared by adults, infants, and non-human animals (Halberda et al., 2008). To date, consensus has yet to be reached regarding the potential relationship between ANS acuity and mathematical skill. Some studies suggest that the ANS serves as a building block for higher mathematical abilities and that ANS acuity facilitates the early learning of symbolic numerical knowledge (e.g., Barth et al., 2008; van Marle et al., 2014). Nevertheless, weakly developed ANS acuity results in mathematical disability (Mazzocco et al., 2011; Geary, 2013). In contrast, others have suggested that innate ANS acuity is not predictive of children’s later mathematical achievement. For example, some studies have revealed that children’s performance on an ANS task is not associated with their math achievement scores (Gilmore et al., 2013; Sasanguie et al., 2013). Given these studies, whether ANS acuity affects arithmetic performance needs to be further verified.

### Study Rationale

The studies outlined above concentrated on domain-general factors or domain-specific factors that affect mathematics and strategy utilization separately. However, studies investigating the effect of the interaction between domain-general and domain-specific factors on math abilities are scarce. Additionally, although studies have shown that ANS acuity is a predictor of mathematics ability, this relationship explained only a small proportion of the total variance. ANS acuity is not the only or the strongest determinant of a child’s math achievement. A theory of early foundations for mathematics learning assumes that the first step toward learning mathematics in children is based on the ANS. The first abstract math symbols are number words and Arabic numerals, which, in turn, obtain their meaning when mapped onto the ANS, during which attention control plays an important role (Geary, 2013). As Iran-Nejad’s wholetheme spiral of biofunctional understanding suggests, multiple sources contribute to learning activities (Iran-Nejad and Irannejad, 2017a). Specifically, the active “I” (e.g., CE, which is energy-depleting, anxiety-inducing and effortful) plays an important role in intuitive understanding (immediate and effortless biofunctional understanding) and conceptual understanding (Iran-Nejad and Bordbar, 2013). Additionally, brain-image evidences show that mathematical intuition and arithmetic performance may emerge from the interplay multiple brain systems. For example, an earlier brain image study found that mathematical intuition depended on visuo-spatial representation and approximate arithmetic recruited bilateral areas of the parietal lobes involved in visuo-spatial processing (Dehaene et al., 1999). The processing of non-symbolic magnitude activated superior parietal cortex related to shifts of visual attention (Sathian et al., 1999; Holloway and Price, 2010). In sum, arithmetic tasks recruits a widespread network of brain regions including the dorsolateral and ventrolateral prefrontal cortex, anterior cingulate, supramarginal gyri, the occipito-ventral cortex and the medial temporal lobe (Peters and De Smedt, 2017). These studies suggest that both ANS acuity and domain-general abilities contribute to arithmetic performance. Moreover, domain-general skills may mediate the relationship between children’s ANS acuity and mathematics ability. Therefore, potential mediating variables in the relationship between ANS acuity and math achievement should be considered.

Given WM and ANS acuity contributions to mathematical ability, we assumed that (1) CE predicts strategy utilization beyond measures of the PL and VS and (2) ANS acuity may play a mediating role in the relationship between CE and strategy utilization.

To date, most studies have focused on the average effect of ANS acuity, PL, and CE on average strategy utilization performance using the ordinary least squares (OLS) regression technique, which can hide important features of the underlying relationship. The regression curve provides a grand summary for the averages of the distribution corresponding to the set of X’s. Thus, ordinary regression often gives a rather incomplete picture, just as the mean gives an incomplete picture of a single distribution (Koenker, 2005). Children who have poor strategy utilization performance tend to be more likely to achieve lower math test scores (Zhang et al., 2014; Si et al., 2016), making it important to explore the determinants of the lower tail and upper tail of strategy utilization performance. Considering the limitations of previous studies on the lower and higher levels of strategy utilization performance, we first introduced quantile regression into psychological research and investigated how WM and ANS acuity affected different levels of strategy performance. Quantile regression computes several different regression curves corresponding to the various percentage points of the distributions and, thus, produces a more complete picture of the set (Koenker, 2005). Quantile regression has been successfully used in a broad range of applications, such as economics, to study determinants of wage (Koenker, 2005) and evaluate value-at-risk models (e.g., Gaglianone et al., 2011; Gerlach et al., 2011). It has also been widely used in the field of environmental health (Bind et al., 2015). In the present study, we expected that the effects of ANS acuity and WM components on strategy utilization would vary according to quantile.

## Materials and Methods

### Participants

In this study, 179 fourth-grade children were selected from public schools in China. Fourth graders were selected because computational estimation problems are frequently introduced in the curriculum at this time. Two children who enrolled in the study were excluded due to the absence of Automated Working Memory Assessment (AWMA) task results, and 17 children were excluded due to an invalid Arithmetic Computational Strategy Test. A total of 160 children were included as study participants (chronological age *M* = 11.09 years, *SD* = 0.65; female = 88). All participants gave written informed consent and were paid for their participation. This study was approved by the regional ethics committee for biomedical research.

### Materials

#### Automated Working Memory Assessment

**FIGURE 1:**
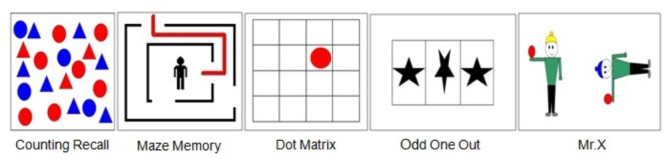
An example of each subtest stimulus of AWMA to measure WM.

The AWMA (Alloway, 2007) was utilized to assess participants’ WM skills. It is a computerized assessment battery of WM skills for individuals 4–22 years of age. Thus, the administration, scoring, and interpretation are fully automated. There are eight subtests to measure three components of WM, such as the digit recall task and word recall task to measure PL (see **Table 1** and **Figure 1** for more details). The test–retest reliability coefficients are presented in **Table 1**.

**Table 1:** The memory subtests, associated memory components and reliability coefficients.

Construct	Subtests	Reliability Coefficients
Phonological Loop	Digit Recall	0.84
	Word Recall	0.76
Visuo-Spatial Sketchpad	Dot Matrix	0.83
	Maze Memory	0.81
Central Executive	Backward Digit	0.64
	Counting Recall	0.79
	Odd One Out	0.81
	Mr. X	0.77

##### Phonological Loop

In Digit Recall, the examinee hears a sequence of digits and attempts to recall each sequence in the correct order. The test begins with a block of one number and increases to a block of nine numbers. Backward Digit is almost identical to Digit Recall in structure and administration; the child hears a sequence of digits and attempts to recall each sequence in backward order. The test begins with a block of two numbers and increases to a block of seven numbers. As the name implies, Word Recall is a series of words that the participants must recall in the correct order. The test begins with a block of one word and increases to a block of seven words.

##### Visuo-Spatial Sketchpad

In Dot Matrix, a red dot in a 4 × 4 matrix is exposed for 2 seconds (s), and then the participants recall the positions by tapping the appropriate squares on a computer screen. The test begins with a block of one dot and increases to a block of nine dots. In Odd One Out, the examinee views three shapes presented in a row of boxes and identifies the Odd One Out shape. After a series of presentations, the examinee must recall the location of each Odd One Out in the correct order. The test begins with a block of one set of shapes and increases to a block of seven sets of shapes. In Maze Memory, the participants view a maze with a red path drawn through it. The task is to use a finger to trace the same path on a blank maze presented 3 s later on the computer screen. The test begins with small mazes and increases to large mazes.

##### Central Executive

In Counting Recall, the child counts the number of red circles in an array of circles and triangles and attempts to recall the tally of numbers in sequence. The test begins with a block of one array and increases to a block of seven arrays of circles and triangles. In Mr. X, the child views a picture of two Mr. X figures and identifies whether the Mr. X with the blue hat is holding the ball in the same hand as the Mr. X with the yellow hat. The Mr. X with the blue hat may also be rotated. At the end of each trial, the child attempts to recall the location of each ball in the correct order by pointing to a picture with six possible marks. The test begins with a block of one set of Mr. X and increases to a block of seven sets of Mr. X.

#### ANS Acuity Task

The Non-symbolic Number Comparison Task is a commonly used measure of ANS acuity. The Weber fraction was used as an index of ANS acuity, as the ANS is assumed to follow Weber’s law (Dietrich et al., 2015). In this task, two sets of dots were presented and participants were asked to determine which of the two sets contained more elements. Visual properties were controlled to ensure that the task actually measured ANS acuity (see Dietrich et al., 2015 for a review); the ratio between two sets of dots was >1.1 but <2.0. Arrays of yellow dots were flashed on a screen for 200 ms, sufficiently short to prevent counting (Mazzocco et al., 2011). The Cronbach’s alpha of the ANS acuity task was 0.80 (He et al., 2016).

#### Arithmetic Computational Estimation Strategy Test (ACST)

The ACST was classified as a measure of an individuals’ strategy utilization, including strategy choice and strategy execution. It consists of 180 two-digit multiplication problems (60 in each of the choice conditions to assess strategy choice, the no choice/rounding-up and no choice/rounding-down to assess strategy execution). The accuracy of each condition, calculated based on the number of correct answers, was considered as he index of the level of strategy choice or strategy execution. Higher accuracy indicates a greater level of individual strategy choice and execution. There were three conditions in this test: the no choice/rounding-down condition, in which all problems must be answered using a rounding-down strategy (i.e., rounding both operands down to the nearest smaller decade, e.g., 28 × 43 ≈ 20 × 40 = 800); the no choice/rounding-up condition, in which all problems must be answered using the rounding-up strategy (i.e., rounding both operands up to the nearest larger decade, e.g., 28 × 43 ≈ 30 × 50 = 1,500); the choice condition, in which one strategy must be selected from rounding-up and rounding-down strategies for each problem to estimate the correct answer as closely as possible. Cronbach’s alpha coefficients for the ACST were 0.919, 0.759, and 0.613 for the conditions of choice, no choice/rounding-up, and no choice/rounding-down subtests, respectively.

Following previous findings (Geary, 1996 for reviews), the following additional constraints were imposed on the selection of problems: (a) no operand included for the digits 0 or 5 (e.g., 30 or 35), (b) no operand included for the same tens digit (e.g., 42 × 46), (c) no operand included for a repeat digit (e.g., 22 × 59), (d) no problem included reversed operands (e.g., if 34 × 87 was used, 87 × 34 was not), (e) half of the larger operands were presented on the right and the other half were presented on the left, and (f) half of the larger units of operands were presented on the right and the other half were presented on the left.

### Procedure

**FIGURE 2:**
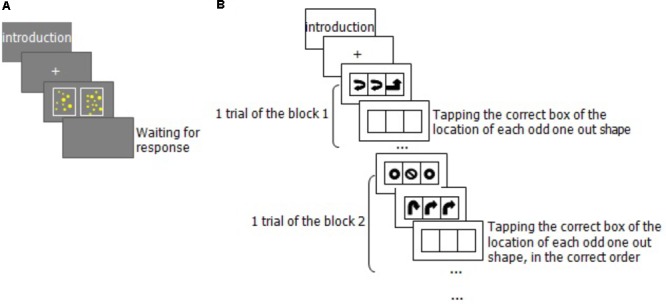
Experimental procedures of ANS acuity task **(A)** and test trials of Odd One Out **(B)**.

Participants were tested in a quiet room. There were two sessions. The first session was a group test, in which participants were asked to complete the ACST. It lasted approximately 15 minutes (min). Before starting the ACST, the experimenter showed each participant how to use the rounding-up and rounding-down strategies. The ACST did not start until every participant could use the strategies proficiently. Participants solved problems according to the instructions. The second session was the individual test, and participants completed the AWMA task first (about 45 min). **Figure 2B** is an example of the Odd-One-Out procedure. The Non-symbolic Number Comparison task was then performed to counterbalance the order across participants (about 10 min, see **Figure 2A**). We scheduled a short break during the testing session.

The AWMA is a computer-based assessment of WM skills. All tests were presented on a laptop computer. The experimenter recorded the participant’s response using the right arrow key on the keyboard (→) for a correct response and the left arrow key on the keyboard (←) for an incorrect response. Each memory test began with a series of practice trials. Immediately following the practice trials were the test trials. The test trials were presented as a series of blocks; each block consisted of six trials. In each case, the child had to remember a piece of information and then recall it back immediately. The participants repeated the information verbally, and the experimenter recorded the participant’s responses to the PL tests (Digit Recall, Backward Digit, and Word Recall). All tests operated on the following rules.

If an individual responded correctly to the first four trials within a block of trials, the program automatically proceeded to the next block. A score of 6 was given for the block that has just been completed.

If an individual responded correctly to four out of five trials in a block, the program immediately started the next block. A score of 5 was given for the block that has just been completed.

If three or more errors were made within a block of trials, the program stopped the test and automatically returned to the main menu. The score for that test reflected the number of correct responses up to the point at which the test was ended.

### Data Analysis

Structural equation modeling (SEM) and quantile regression techniques were used to analyze the data with AMOS 17.0 (IBM/SPSS Inc., Chicago, IL, United States) and SAS 9.4 (SAS Institute, Cary, NC, United States), respectively.

## Results

In this section, we present our results that (a) compare the predictions of PL, VS, and CE for children’s strategy utilization, (b) demonstrate the role CE played in the relationship between children’s ANS acuity and strategy utilization, and (c) resolve the different effects of WM and ANS acuity on lower-, middle-, and higher-level strategy utilization performance.

### Preliminary Analyses

**FIGURE 3:**
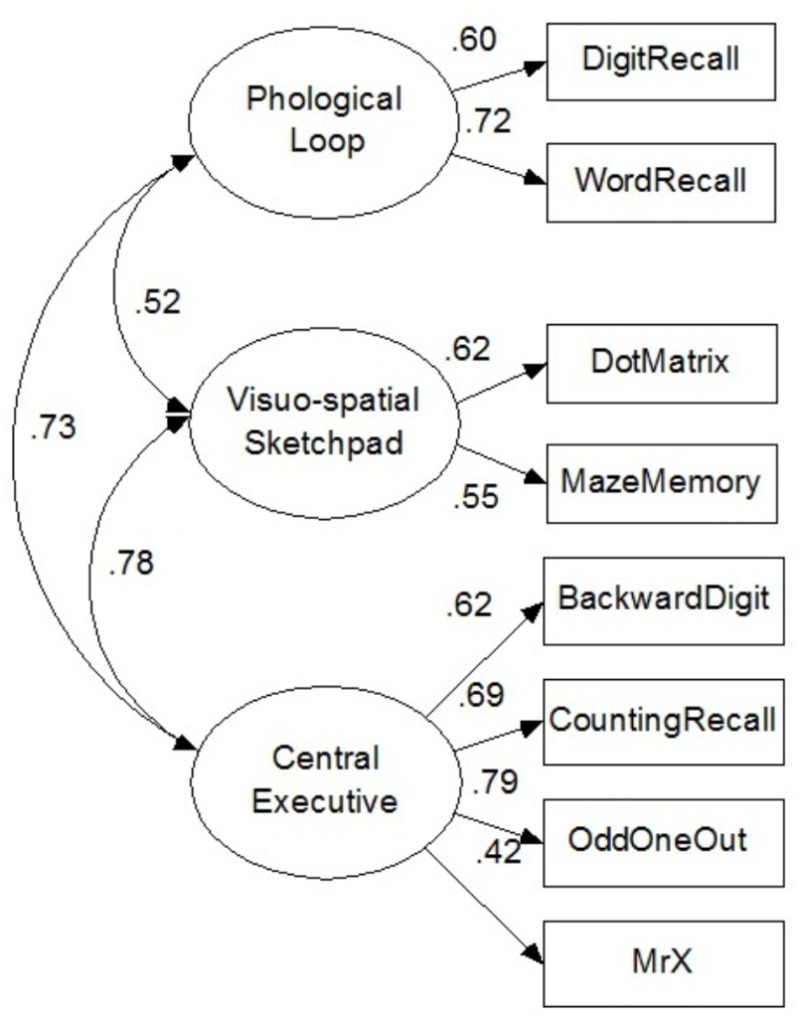
Measurements model testing the Baddeley Multicomponent Model. χ^2^ = 18.56, *df* = 17, *p* = 0.36, RMSEA = 0.02, CFI = 0.98, TLI = 0.99.

A first-order confirmatory factor analysis was used to determine whether the data acquired by the AWMA fit with the WM theory model and SEM using maximum likelihood estimation in AMOS 17.0. The model demonstrated a good fit to the data: χ^2^ = 18.56, *df* = 17, *p* = 0.36, RMSEA = 0.02, GFI = 0.97, TLI = 0.99 (see **Figure 3**). All structural path coefficients were significant (*p* < 0.001, βs, 0.4–0.8).

Means, standard deviations, and correlations for the variables are shown in **Table 2**. Consistent with previous research on children of this age (Pizza et al., 2010), this study revealed that children’s ANS acuity (average *w*) was 0.215. The Weber fraction as an index of ANS acuity is inversely proportional to ANS acuity. Thus, to facilitate the description, *w* was multiplied by -1. As expected, ANS acuity was significantly related to PL, VS, and CE, with a particularly strong relationship between ANS acuity and CE (*r* = 0.39). Children’s performance on ANS acuity was also significantly associated with their strategy utilization performance. Consistent with a prior study, children’s PL, VS, and CE were significantly correlated with strategy utilization, with a particularly strong relationship between CE and strategy use (*r* = 0.50).

**Table 2:** Descriptive statistics for all measures (*M* and *SD*) and correlation among variables.

Measures	1	2	3	4	5	6	7
(1) ANS A (*-w*)	1						
(2) PL	0.27^∗∗^	1					
(3) VS	0.27^∗∗^	0.28^∗∗^	1				
(4) CE	0.39^∗∗^	0.50^∗∗^	0.48^∗^	1			
(5) SC	0.35^∗∗^	0.26^∗∗^	0.28^∗∗^	0.47^∗∗^	1		
(6) SE	0.30^∗∗^	0.31^∗∗^	0.19^∗^	0.32^∗∗^	0.37^∗∗^	1	
(7) SU	0.38^∗∗^	0.31^∗∗^	0.30^∗∗^	0.50^∗∗^	0.97^∗∗^	^∗∗^	1
*M*	0.21	121.72	93.55	110.45	74.44	98.161	90.254
*SD*	0.09	13.12	13.80	12.74	16.39	2.45	6.25

*ANS A, approximate number system acuity; PL, phonological loop; VS, visuo-spatial sketchpad; CE, central executive; SC, strategy choice; SE, strategy execution; SU, strategy utilization. ^∗^*p* < 0.05, ^∗∗^*p* < 0.01, ^∗∗∗^*p* < 0.001, similarly hereinafter.*

The independent sample *t*-test was used to test the gender differences in the variables. No significant gender differences were found for different components of WM (PL: *t* = -1.21, *p* > 0.05; VS: *t* = 1.81, *p >* 0.05; CE: *t* = -1.35, *p* > 0.05), ANS acuity (*t* = 0.06, *p* > 0.05), or strategy utilization (strategy choice: *t* = -1.47, *p* > 0.05; strategy execution: *t* = -0.44, *p* > 0.05); therefore, we built only one SEM without differentiating gender.

### Structural Equation Model Analyses

Three SEM analyses using a maximum likelihood estimation were performed. First, two direct effects models were prepared, where the direct effects of PL, VS, and CE on strategy choice (Model 1) and strategy execution (Model 2) were tested separately. These two models indicated a good fit to our data (see **Table 3**).

**Table 3:** Fit statistics on the Structural Equation Models and the corresponding fit criteria.

Models	Fit indices						
	χ^2^	*df*	χ^2^/*df*	CFI	TLI	RMSEA	*p*
Model 1	34.36	29	1.19	0.98	0.97	0.034	0.23
Model 2	35.98	22	1.64	0.96	0.93	0.06	0.03
Model 3	24.42	17	1.44	0.97	0.95	0.05	0.11
Fit criteria		
Acceptable fit			≤5.00	≥0.90	≥0.90	<0.08	>0.01
Good fit			0.00 ≤ χ^2^/*df* ≤ 2	≥0.95	≥0.95	<0.05	

Models 1 and 2 show the direct effects of PL, VS, and CE on strategy execution and strategy choice, respectively. The results indicated that CE highly predicted individual differences in strategy choice (β = 0.83, *SE* = 0.78, *p* = 0.03) beyond the effect of strategy execution (β = 0.42, *SE* = 0.21, *p* = 0.38). PL also positively influenced strategy executive (β = 0.27, *SE* = 0.05, *p <* 0.05), but negatively affected strategy choice (β = -0.16, *SE* = 0.23, *p* > 0.05). Moreover, although they were not statistically significant, VS negatively predicted children’s performance on strategy execution (β = -0.17, *SE* = 0.01, *p* > 0.05) and strategy choice (β = -0.10, *SE* = 0.48, *p* > 0.05).

**FIGURE 4:**
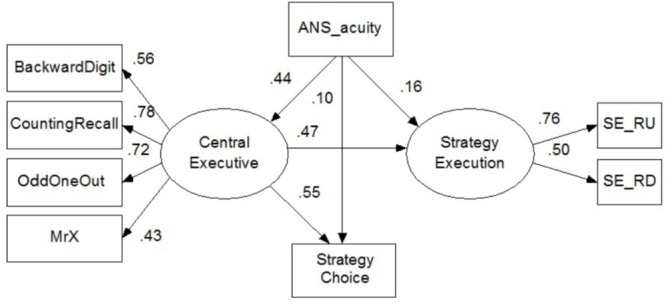
The complete mediation model of effects of CE on strategy utilization. In the depicted model, 30.8 and 36.5% of the variance of strategy execution and strategy choice were explained. χ^2^ = 24.42, *df* = 17, *p* = 0.11, RMSEA = 0.05, CFI = 0.97, TLI = 0.95.

Given that the effect of CE on strategy utilization was much stronger than that of PL and VS, we tested the complete mediation model, which assumed that CE completely mediated the effects of ANS acuity on children’s strategy utilization, specifically in strategy choice and strategy execution. This model demonstrated good fit, χ^2^ = 24.42, *df* = 17, *p* = 0.11, CFI = 0.97, TLI = 0.95, RMSEA = 0.05. All structural path coefficients were significant (βs, 0.2–0.6, *p* < 0.01), with the exception of the non-significant path for the relationship between ANS acuity and strategy utilization (strategy execution: β = 0.06, SE = 0.12, *p* = 0.20; strategy choice: β = 0.10, *SE* = 0.08, *p* = 0.23, see **Figure 4**). With this model structure, 30.8 and 36.5% of the variance of children’s strategy execution (*R^2^* = 0.31) and strategy choice (*R^2^* = 0.37) were explained, respectively.

As expected, the strategy execution results revealed that ANS acuity significantly predicted children’s performance on strategy execution (β = 0.37, *SE* = 0.11, *p* = 0.001), but it was not significant after controlling for CE (β = 0.16, *SE* = 0.12, *p* = 0.20). Similarly, the strategy choice results showed that ANS acuity significantly predicted children’s performance on strategy choice (β = 0.35, *SE* = 0.07, *p* < 0.001), but ANS was not a significant predictor when controlling for CE (β = 0.10, *SE* = 0.08, *p* = 0.23). These results suggested that the associations between ANS acuity and strategy execution and ANS acuity and strategy choice were completely mediated by CE; the mediating effects accounted for 56.3 and 70.5%, respectively, of the variance in children’s performance on strategy execution and strategy choice separately.

### Quantile Regression Analyses

Quantile regression analyses were used to test our hypothesis that ANS acuity and WM components generate different effects on low-level and high-level strategy utilization performance. In this study, SEM analyses revealed that ANS acuity and WM performance only accounted for a small proportion of the variance in strategy execution; thus, we investigated the determinants of the logarithm of children’s strategy choice based on their ANS acuity, PL, and CE. The results are presented in **Table 4** for five distinct quantiles. In the last three rows of the Table, the conditional mean effect estimated by least squares is reported. A number of interesting findings were revealed.

**Table 4:** Quantile regression and ordinary least squares parameter estimates.

	Parameter Estimates					
Quantile	Parameter	Estimate	*SE*	95% Confidence Limits		*t*
0.15	Intercept	–21.29	29.78	–80.29	36.43	–0.74
	ANS Accuracy	38.20	31.38	–99.71	23.30	1.04
	Phonological Loop	0.00	0.17	–0.33	0.33	0.03
	Central Executive	0.80	0.18	0.45	1.15	449^∗∗∗^
0.25	Intercept	–15.27	25.90	–66.04	35.50	–0.59
	ANS Accuracy	32.61	30.26	–91.93	26.71	1.02
	Phonological Loop	–0.04	0.18	–0.40	0.32	–0.24
	Central Executive	0.86	0.19	0.48	1.23	4.44^∗∗∗^
0.5	Intercept	20.47	15.90	–10.70	51.63	1.29
	ANS Accuracy	46.68	11.87	–70.96	–23.41	4.27^∗∗∗^
	Phonological Loop	0.04	0.19	–0.33	0.40	0.20
	Central Executive	0.55	0.15	0.27	0.85	3.67^∗∗∗^
0.75	Intercept	62.46	14.77	33.51	91.41	4.23
	ANS Accuracy	37.81	13.05	–63.39	–12.24	4.14^∗∗^
	Phonological Loop	–0.03	0.12	–0.27	0.21	–0.24
	Central Executive	0.31	0.12	0.08	0.54	2.66^∗∗^
0.85	Intercept	79.07	14.37	50.94	107.2	6.61
	ANS Accuracy	36.56	16.20	–66.34	–6.77	2.41^∗^
	Phonological Loop	–0.11	0.12	–0.35	0.14	–0.87
	Central Executive	0.27	0.12	0.03	00.50	2.24^∗^
OLS						
	ANS Acuity	34.29	13.55			2.53^∗^
	Phonological Loop	0.019	0.10			0.19
	Central Executive	0.50	0.11			4.68^∗∗∗^

**FIGURE 5:**
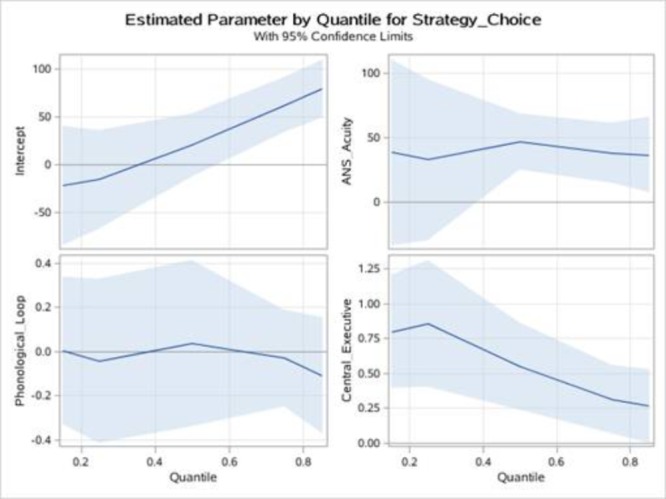
ANS acuity, PL and CE of strategy choice: quantile regression estimates of the strategy choice. ANS acuity, PL and CE are plotted as a function of the quantile of the strategy choice distribution. Shadow represents 95% confidence band.

It was clear that the OLS estimates do not tell the whole story. However, the quantile regression curves show that the value of the estimated coefficient on ANS acuity, PL, and CE varied over the conditional growth rate distribution, which was consistent with our prediction. In particular, at the 0.15 and 0.25 quantiles, the estimate of ANS acuity was insignificant, that is, ANS acuity did not affect the low-level strategy choice. ANS acuity was significantly associated with strategy choice at the 0.5, 0.75, and 0.85 quantiles. Namely, ANS acuity was of crucial importance for median and higher than median strategy choice performance. Moreover, the CE estimate was significant in all quantiles. In contrast, PL was not significantly associated with strategy choice. Note that when the quantile regression solution was evaluated at the lower-level of strategy choice performance (i.e., at the 0.15 and 0.25 quantiles), CE appeared to have a large effect on children’s strategy choice performance. However, the CE coefficient decreased sharply for higher-level strategy choice performances at the upper quantiles, suggesting that the effect of CE decreased as strategy choice performance increased (see **Figure 5**).

## Discussion

This study explored the complex associations between ANS acuity, WM, and strategy utilization. In the following paragraphs, we first discuss a potential account of the underlying mechanism for the relationship between ANS acuity and strategy utilization. We then elaborate on how ANS acuity and WM components affected strategy choice performance in the tail of the distribution. Finally, we propose a possible explanation for the relationship between WM components and strategy utilization.

Although some studies show that ANS acuity predicts mathematical ability, this relationship explains only a small proportion of the total variance. In fact, previous studies have reported that individual differences in ANS acuity correlate with variance in cognitive skills. How ANS acuity relates to other factors that affect math ability is unknown. In the present study, the mediation model was used to explore how ANS acuity influenced children’s strategy utilization (strategy execution and strategy choice) through CE. Our results showed that CE completely mediated the relationship between ANS acuity and strategy utilization. This was in line with Hassinger-Das et al. (2014) who found that both executive functioning and attention problems partially mediate the relationship between number sense in kindergarten and mathematics achievement in the first grade. These results suggest that domain-general abilities may play a crucial role in the association between children’s mathematics abilities, which was an important finding in the study. The mathematics learning model developed by Geary (2004, 2013) is useful for explaining such a mediating effect. This model proposes that mathematics ability relies on a conceptual understanding of procedural knowledge that supports problem solving. Conceptual and procedural abilities, in turn, receive the support of cognitive systems, such as CE. CE controls attention and inhibition and plays a key role in problem solving. Later, Geary (2013) expanded his model by introducing ANS. This model assumes that ANS is the underlying foundation of early symbolic math abilities. In other words, “the initial step toward learning mathematics may be based on an intuitive sense of the approximate magnitude of collections of items. The first abstract mathematical symbols that children learn are number words and Arabic numerals, which, in turn, acquire meaning when mapped onto this number sense” (Geary, 2013). During these processes, WM plays a major role maintaining goal-relevant information and ignoring irrelevant internal distractions and external distractions. This model is inline with Iran-Nejad’s wholetheme spiral of biofunctional understanding. Iran-Nejad and Irannejad (2017a) also elaborated the crucial role of active executive function component in the relationship between intuitive understanding and conceptual understanding. That is to say, both domain-general skills and domain-specific skills contribute to strategy utilization. Therefore, future research needs to explore additional potential mediating variables when studying the relationship between ANS acuity and math ability. From an educational point of view, these findings suggest that we should pay attention not only to cultivation of basic mathematics ability, but to the cultivation of domain-general abilities such as WM, spatial skills.

Another significant finding in the present study was that we first detected the effects of WM components and ANS acuity on strategy choice performance in the tail distribution using the quantile regression technique. The results revealed that the association between CE and strategy choice was robust from the first quantile to the highest quantile. That is, CE contributed not only to low-level strategy choice performance but also to high-level performance. Note that the relationship between ANS acuity and strategy choice was significant at the median and higher quantiles (i.e., 0.5, 0.75, and 0.85 quantiles), but not at lower quantiles (i.e., 0.15 and 0.25 quantiles), meaning that children with low-level strategy choice performance were not affected by ANS acuity, but when children’s strategy choice performance was at the median or far above the median performance, their strategy choice was remarkably affected by ANS acuity. These findings cannot be observed using traditional OLS regression. One possible explanation for this phenomenon is the two-factor theory (Robinson et al., 2002). Robinson et al. (2002) suggested that either weakness in phonological processing or weakly developed ANS acuity may result in mathematics disability. Furthermore, a child who has weaknesses in both experience more learning impediments than a student with only one or the other weakness or with neither weakness at all, as there would not be a compensatory channel available for the child to build upon as a strength. Furthermore, Iran-Nejad’s wholetheme spiral of biofunctional understanding (Iran-Nejad and Irannejad, 2017a) is consistent with the above theory. Iran-Nejad and Irannejad (2017a) proposes that children lacking intuitive understanding (the missing biofunctional component) will compensate by resorting to the active executive function component. In the present study, children with low-level strategy choice performance were not affected by ANS acuity but were strongly affected by CE, suggesting that a child who has poor strategy choice performance may have weaknesses in ANS acuity (Mussolin et al., 2014). Thus, they use CE as a compensatory channel to address the problems. Hassinger-Das et al. (2014) also revealed that children with poor number sense rely heavily on WM to solve math problems. The mutual inclusion/exclusion theory also benefits to understand these results. This theory assumes that if two types of processes need common elements, they may be not co-occur (Diener and Iran-Nejad, 1986; Iran-Nejad and Zengaro, 2013). For example, if one type of affect is at low levels, the other type of affect can be at any level of intensity from low to high (i.e. mutual inclusion). One does not simultaneously experience both positive and negative affect at high level intensity (i.e. mutual exclusion). Similarly, in the present study, our results found that at low level strategy choice, the effect of CE was significant but ANS acuity not. At high level strategy choice, both the effect of CE and ANS acuity were significant. That is, ANS acuity plays an important role in individual with middle and high level strategy performance. According to mutual inclusion/exclusion theory, we assumed that participants with high level strategy choice also possess high ANS acuity (Halberda et al., 2008; Starr et al., 2013), high ANS acuity and CE together promote problem solving(mutual inclusion). However, participants with low ANS acuity may experience high mathematics anxiety (dynamic self-regulation) (Lindskog et al., 2017), which does not benefit to solve arithmetic problems (mutual exclusion). These findings are of immense help to math education. Specifically, teachers can teach students in accordance with their aptitudes. Additionally, children with poor mathematics performance or mathematics difficulties are likely to have impaired basic mathematics skills. Brain image studies also support these findings. Namely, children with lower mathematical skills show increased activity in numerical magnitude processing related areas of the arithmetic network (see Peters and De Smedt, 2017, for a review). There exists evidence that exercise of basic numerical processing skills or non-symbolic numerical process can improve children’s mathematics performance (Obersteiner et al., 2013; Hyde et al., 2014). Thus teachers should pay more attention to children who perform poorly in mathematics and take interventions to train their basic mathematics ability, ultimately improve their mathematics performance.

An unexpected additional interesting finding that contrasts with previous studies was the insignificant relationship between VS WM and strategy utilization. As discussed by Belacchi et al. (2014), children’s success on an arithmetic task is linked to success on WM tasks, particularly the VS component. Similarly, Kim and Cameron (2016) also revealed that weak VS sketchpad skill is detrimental for mathematics development and that strengthening weaker VS sketchpad skills benefits children’s mathematics learning. How can we account for these inconsistent findings? The first hypothetical explanation refers to the different tasks used in various studies. Visual-spatial skill is the response of short-term storage of visual and spatial information, such as memory for objects and their locations. CE is analogous to an executive board that controls attention, selects strategies, and integrates information from several different sources. In the present study, strategy utilization was measured using arithmetic problems (e.g., 28 × 43 = ?). Belacchi et al. (2014) used the approximate addition task (i.e., dots comparison) to assess math ability. This task asks participants to estimate the numerosity of blue dots obtained after adding two different sets together in comparison with a reference set. It is well known that the locations of dots presented on a screen were random and that the participant needed to remember the spatial locations of dots, which requires more visual resources to capture stimulating information. However, in our study, problems were presented at the center of the screen; thus, participants did not need extra VS WM. That is to say, the task in our study required more CE rather than VS skills. Previous studies also reported that CE is a link to strategy use (Bull and Lee, 2014; Blair et al., 2015), problem solving (Best et al., 2011), and simple calculations (Fuchs et al., 2010), but VS was linked to spatially represent and interpret numerosity information (Geary, 1993; Gunderson et al., 2012) and an approximate calculation and estimate (Gunderson et al., 2012). A second possible explanation is related to the participants. The two-factor theory suggests that when a child is weak in one skill, they draw on additional skills as a compensation mechanism to solve math problems (Robinson et al., 2002). Individuals with Down’s syndrome (Rowe et al., 2006) or autism and William’s syndrome (Kim and Cameron, 2016) perform at a significantly lower level on tests of execution. The Down’s syndrome group also performs poor in PL (Rowe et al., 2006). Thus, we postulate that individuals defective in CE or PL may rely heavily on VS to solve arithmetic problems. However, normally developing children whose CE was not impaired in our study still drew heavily on CE to finish arithmetic tasks rather than resorting to VS. Of course, future research should verify these hypotheses.

On a theoretical level, the present findings have significant implications regarding our understanding of strategy selection. The strategy choice and discovery simulation model (SCADS) assumes that there is a WM trace of each strategy’s execution, which allows individuals to provide accurate information immediately after verbal reports concerning their strategy use (Shrager and Siegler, 1998). Consistent with the SCADS, the present data provide further empirical evidence for the effect of WM on strategy use. Our data also demonstrate that ANS acuity is related to strategy utilization. Indeed, ANS acuity affected children’s strategy utilization through CE. Two studies found a causal association between ANS acuity and math ability (Park and Brannon, 2013; He et al., 2016). Impaired acuity of the ANS underlies dyscalculia (Mazzocco et al., 2011), which has lifelong consequences on outcomes such as job attainment and decision making (Hibbard et al., 2007). Therefore, we suggest that SCADS needs to be considered in the relationship between ANS acuity and strategy utilization, the mediating effect of CE on them, and individual differences in strategy utilization performance.

Although our results support our hypotheses, there were at least two additional limitations of this study that suggest directions for future studies. First, we recruited typically developing children in this study. As discussed above, the effect of ANS acuity and WM components on strategy choice varies from the 0.15 to 0.85 quantiles. Accordingly, future research should pay more attention to children who perform in the tail of the distribution. Second, we “left out critical co-requisite sources of contribution, something that happens all too often in both psychology and education” (Iran-Nejad and Irannejad, 2017b). According to active-dynamic theory (Iran-Nejad, 1990; Iran-Nejad and Chissom, 1992), CE, as an active source of learning, can influence learning and performance through dynamic self-regulation (Iran-Nejad and Homaifar, 2005). However, in the present study, we mainly investigated how CE contribute to arithmetic performance and didn’t explore the effect of dynamic source on it. Although this study result was supported by some theory (Geary, 2004, 2013) and empirical studies (Hassinger-Das et al., 2014; Bull et al., 2017), we cannot ignore the role of dynamic source. Moreover, dynamic interest-creating discovery modules are highly alterable (Iran-Nejad and Chissom, 1992), which is important to educational intervention. Future research should explore the effect of dynamic source on the relationship between CE and arithmetic performance.

## Ethics Statement

All procedures performed in studies involving human participants were in accordance with the ethical standards of the ethics committee on human experimentation of Shandong Normal University and with the 1964 Helsinki declaration and its later amendments or comparable ethical standards.

## Author Contributions

Conception and design of the work were done by HL and JS. Data collection was done by HL, MZ, XW, and XD. Analysis of data was done by HL, JS, and all other authors interpreted the data. The original draft of the manuscript was wrote by HL, and it was edited and co-wrote by MZ, XW, XD, and JS.

## Conflict of Interest Statement

The authors declare that the research was conducted in the absence of any commercial or financial relationships that could be construed as a potential conflict of interest. The reviewer FB and handling Editor declared their shared affiliation.
